# Notes and Queries

**Published:** 1895-05-18

**Authors:** 


### NOTES AND QUERIES.

Mr. A. C. Jackson, M R.C.S.E., L.P.C.P. Ed., L.M. Lond. L.M. Ed., writes from 257, Finchley Road, N.W.: Kindly inform me (if possible) as to the probability of obtaining information respecting any appointments as medical officer in any colonial hospital. I have just completed a three years' engagement at one and am desirous of re-engagement.

*** Mr. Jackson had better call at the offices of the various Agents General, most of which are situated in Victoria Street, S.W.

